# Family Health Conversations: How Do They Support Health?

**DOI:** 10.1155/2014/547160

**Published:** 2014-04-01

**Authors:** Carina Persson, Eva Benzein

**Affiliations:** Department of Health and Caring Sciences, Faculty of Health and Life Sciences, Center for Collaborative Palliative Care, Linnaeus University, 391 82 Kalmar, Sweden

## Abstract

Research shows that living with illness can be a distressing experience for the family and may result in suffering and reduced health. To meet families' needs, family systems intervention models are developed and employed in clinical contexts. For successful refinement and implementation it is important to understand how these models work. The aim of this study was therefore to describe the dialogue process and possible working mechanisms of one systems nursing intervention model, the Family Health Conversation model. A descriptive evaluation design was applied and 15 transcribed conversations with five families were analyzed within a hermeneutic tradition. Two types of interrelated dialogue events were identified: narrating and exploring. There was a flow between these events, a movement that was generated by the interaction between the participants. Our theoretically grounded interpretation showed that narrating, listening, and reconsidering in interaction may be understood as supporting family health by offering the families the opportunity to constitute self-identity and identity within the family, increasing the families' understanding of multiple ways of being and acting, to see new possibilities and to develop meaning and hope. Results from this study may hopefully contribute to the successful implementation of family systems interventions in education and clinical praxis.

## 1. Introduction

A substantial body of research reports of illness related distress and its impact on quality of life for the person living with illness as well as for their significant others (i.e., spouses and family caregivers) is available. Most of these studies focus on the individual. There are, however, few studies that focus on the family system and describe illness experiences from the perspective of the family as a unit. These studies show that living with illness is experienced as family vulnerability, helplessness, strain, and suffering [1], as a struggle to make sense and maintain normality in family living [2] and as bringing about difficulties in family relationships [3].

The results from the above reported system focused studies point to a need to expand the focus for care in order to support families living with illness and also to broaden the concept of health to embrace the family as a unit, that is, family health. Family health has been described as an interactional, holistic, and dynamic phenomenon positing circular causality [4] and comprising biological, psychological, spiritual, sociological, and cultural aspects of well-being both on an individual and family level [5]. The theoretical ground for this understanding is system theory which focuses on interactions among the various parts of a system and the system as a whole [6]. One example of this systemic view is Newman's theory of health as expanding consciousness in which individuals are seen as open systems that constantly interact with their environment. One family member's illness is reflected in the pattern of family interaction and when these patterns are made conscious, health will be gained [7].

Although research findings and system theories suggest that chronic illness has an impact on the family as a unit, the health care system is typically patient focused and family systems needs are notably unmet. Recently published reviews of family interventions [8–14] show that interventions provided by health care often are characterized as psychoeducational versus relationship focused. Additionally, interventions are mostly directed towards the individual family member or towards the partner dyad. Outcomes targeting individual outcomes are domineering. Responding to families' experiences of distress and the lack of systems oriented support from health care, family systems interventions have developed within the nursing profession.

The majority of family systems nursing interventions are grounded in the so-called Calgary models, Calgary Family Assessment Model and Calgary Family Intervention Model [15], and in the Illness Belief Model [16]. The aim of these interventions is to support family health by creating a context for change [16]. This context is facilitated in the interactions between the participants by assessing the families' situations and intervening by posing reflective questions targeting the problems experienced by the families [15]. Changes that are hoped for concern the families' ways of thinking and responding in relation to the illness situation and to those problems experienced, having impact on their well-being. This may embrace change or modification of constraining beliefs and strengthening of facilitating beliefs, of finding alternative ways of talking about the family situation and the discovery of new meanings [15, 16]. A small but growing body of qualitatively designed outcome studies focusing on families' experiences points at these interventions to be a healing experience [17], improving family relationships [18, 19], alleviating experiences of suffering [20, 21], and psychologically empowering [22, 23].

For the development of family systems support with the purpose to improve and promote family health when living with illness, knowledge about the nature of the intervention processes and understanding of the potent working mechanisms are central. Most of the interventions are theoretically grounded but have not been evaluated from how they are practiced. Evaluations that focus on the processes that actually take place can contribute with knowledge of the working mechanisms in the intervention [24]. This type of knowledge is a prerequisite for successful refinement and implementation in health care. Therefore, the aim of this study was to describe the dialogue process and possible working mechanisms of the Family Health Conversation (FamHC) [18]. The research questions were as follows. What dialogue events may be identified and how are they interrelated? What characterizes the interactions between the participants? How may the events and interactions be understood as supporting family health?

## 2. Research Design and Methods

### 2.1. Research Design

An evaluative approach aiming at a description of the intervention process and the working mechanisms of this process [24] was chosen. Data consisting of transcripts from the interventions were analyzed within a hermeneutic tradition that emphasize the necessity to use ones pre-understanding in the interpretation process but still remain open to the phenomenon at hand. Openness is supported by not using theories in this phase of the analysis and also by getting into a “dialogue” with the text in order to reach a first understanding. This understanding should be critically examined in relation to the text throughout the analysis [25–27]. By going into the hermeneutical spiral, where the parts are considered in relation to the whole and vice versa, the interpretations can be validated so that the best possible interpretation is reached [26, 27], an understanding that is grounded in a fusion of horizons [25]. To gain a deeper understanding of the phenomenon, the findings could be further reflected on in relation to theories or philosophical reasoning [26, 27].

### 2.2. Intervention

The Family Health Conversation Model was developed by the last author and her colleagues at the Linnaeus University in Sweden during the late 1990s [18]. The model is inspired by the Calgary Family Assessment and Intervention Models [15] and particularly the Illness Beliefs Model [16]. The conversation model builds on system oriented theories and models [6, 15, 28], change theory [29], and narration and reflection theory [26, 30, 31]. The model is used by the research team in clinical practice and in research [17, 18]. The structure of the model is three conversations, a closing letter, and an evaluative follow-up interview. The three conversations have somewhat different intentions [18]. The first conversation with each family started with a discussion of the aims and of the family members' expectations of how the conversations possibly could support them. Then, each family member was invited to tell their story about how they experienced the family's situation. Based on these stories, the family and the two conversational leaders together agreed what to talk about and what changes might be desirable and possible for the families. The intention in the second conversation was to progress further towards minimizing the family's suffering by illuminating family members' beliefs and by strengthening facilitating beliefs and modifying constraining beliefs. The intention of the third conversation was much the same as the second but also included a termination of the series of meetings and a reflection of the process of change that had occurred.

### 2.3. Data

Data for this study consisted of 15 transcribed FamHCs with five families that had participated in a series of three conversations. Each series was held within a period of 6–10 weeks. The time for each conversation varied between 60 and 80 minutes.

### 2.4. Participants

A purposive sampling strategy was used to get a sample of families that varied according to chronic illness, time since onset, sex, and age of patients and family members. The five families that were included had all participated in FamHC either at our campus-based Center for research on families' health (*n* = 3) or in their own homes (*n* = 2). They were either self-referred or recruited from a rehabilitation clinic at the local hospital (Table 1). Four nurses from the research team, of whom one is the second author of this paper, participated in three different pair constellations of conversational leaders. All nurses had education at advanced level and several years of experience with this type of conversations.

### 2.5. Ethical Considerations

This study was approved by the Research Ethics Committee at Linköping University, Sweden (reference number 2010/51-31) and was conducted in accordance with the Declaration of Helsinki (1964). Participants gave their written consent for the tape-recorded conversations to be used for research purposes. Efforts to preserve confidentiality were guaranteed.

### 2.6. Analysis

The analysis comprised two phases in relation to the use of theories: one inductive phase and one deductive phase. In the inductive phase, dialogue events were described and explored by the first author (Carina Persson) who was not yet familiar with the intervention model or its central assumptions and theories and thereby data could speak “for itself.” The transcribed conversations were read, one conversation at a time, and text segments focusing on dialogue events were identified. The identified text segments were reread focusing on the question: what are the characteristics of these dialogue events? One further reading was done to identify sequence patterns within and between the dialogue events guided by the question: what is preceding and what follows? The author strived to become involved in a hermeneutical “dialogue” [25, 26], that is, asking new questions to the text, gaining new understanding, and asking new questions, “is it really so? Could it be in any other way?” (see Table 2 for one example of the analysis' process). In the deductive phase, we wanted to further understand how the events and sequence patterns may support family health from a theoretical perspective. The results of the inductive analysis were reflected on using literature embracing health theory, systems intervention models, and philosophic literature highlighting phenomenon/concepts with meaning for health.

## 3. Results

### 3.1. Results from the Inductive Phase of the Analysis

Overall, we found that family members narrated and explored the families' concerns in interaction with the conversational leaders. We identified two types of dialogue events characterized as narrating or exploring. We found these to be interrelated, but distinctly identifiable. Usually, a narrating episode flowed into exploring and vice versa. The participants' verbal actions were also analyzed separately, but it was obvious that a reciprocal pattern of actions-responses took place that generated the movement between the two dialogue events (see Table 3).

Narrating episodes were characterized by their descriptive constituent and comprised the families' experiences of living with illness. Family members' reactions, behaviors, and feelings in various daily situations and consequences of these were in focus. The narratives also comprised “simple” explanations of why problematic situations occurred. One example of this is illustrated in Table 3 where the couple narrates their experiences of the woman's difficulties getting ready in time due to her illness. The partner supports the woman's narration by adding his perspective and his explanation of why she does not manage: she finds out other things to do and is not focused on what has to be done. At the end of this event the woman acknowledges the husband's belief that she should focus on one thing at a time and accept her situation. Earlier and also later in the conversation it is clear that the couple are distressed and that the issue has grown into a conflict between them. Not being able to do things together has also contributed to having less in common and a sense of living their lives separate from each other. Families constructed a collective narrative where family members took turns and acted as the “primary” or “secondary” narrator. The primary narrator initiated and took the main responsibility for the narration while the “secondary” narrator spontaneously commented, added to, confirmed, or questioned the other's story. These functions were evident and changed between all of the participating family members and were related to the issues brought up during the conversations. Table 3 illustrates how the woman acts as a primary narrator brings up the problematic issue and takes responsibility to move on. Her partner acts as the second narrator, adding his viewpoint and confirming her difficulties. In the conversations the “primary narrator” also took the opportunity to invite other family members to contribute to the narrative and sometimes narrate on behalf of the “primary narrator.” The latter occurred when the “primary narrator” found it too emotionally upsetting to narrate, or when he/she had memory or communication problems. One example of this is the following citation from one of the families where the woman finds it too upsetting to tell herself.

*Woman with illness: “I've become rather tired the last couple of months. It's been like a trauma between me and my son's wife. She has forbidden me to meet my granddaughter who has turned one and a half. Please can you tell all about it? (turns to her partner), I can't go through it, I can't tell, I won't tell, no” (starts to cry).*

The narrating episodes were initiated by an invitation from conversational leaders and only rarely by another family member (see Table 3 for one example). In the initiating phase, mainly open questions were asked (“How do you think then? In what way…?”). “Statement questions” (“Are your opinions…? Do you then think that…?”) linked to the narrative were also frequent. If the family member's response was brief, the conversational leaders asked follow-up questions, made short one-syllable comments, or asked clarifying questions. If a “secondary” narrator had contradictory experiences or opinions, a discussion took place before the narration was resumed or preceded into an exploring episode.

We found that the exploring episodes comprised the family members' reconsiderations and reflections on the situations in the narrating episodes. The participants examined the nature of the situations and discussed possible explanations. From the illustration in Table 3, it is evident that the woman reconsiders the exploring question from the conversational leader that connects the previously narrated problematic issue to the belief that despite the illness, she expects herself to act as before the onset. The family members' various reactions, behaviors, and beliefs were made visible and were also reconsidered in relation to those of other family members and to their impact on family relations. Family members cocreated alternative explanations to why problematic situations occurred and reconsidered their own options to influence and take control over certain situations. Family members' understandings of various situations were reconsidered and sometimes altered, resulting in the development of a new communal understanding of the families' experiences. In Table 3 this is illustrated when the woman expresses a different interpretation of how the problematic issue may be understood. She concludes that you cannot expect to be as before and that she should accept that her illness limits her ability. Her partner can also see that this belief contributes to distress and brings about physical symptoms. The couple expresses a renewed and shared understanding of how the problematic issue may be understood. The interaction pattern was different from that of the narrating episodes. Here, the conversational leaders took the initiative and facilitated exploration and family members responded by means of reconsideration and reflection. The conversational leaders asked questions, for example, when a problematic situation had been narrated or when different experiences or beliefs between family members were discerned. Exploring questions were based on previous narratives. The initiating questions could be “comparative questions”, “analyzing questions,” or “connecting questions” (see Table 4). We also found that follow-up questions were frequently asked in the exploring episodes. In addition, we identified “interpretive questions” and “concluding statement questions” (see Table 4). In Table 3 the conversational leader initiated the exploring episode by posing a comparative question “has it always been like this?” and goes on by posing other comparative questions related to what has previously been narrated by the family members. Finally the conversational leader A poses an interpretive question “yes, do you demand too much of yourself?” The conversational leaders also offered the family the opportunity to listen to their reflections. The family could then choose to bring these into their further exploration. Family members sometimes asked questions. This was most prominent when a family member had difficulties expressing him-/herself, for example, in cases of memory or expressive dysfunctions and when younger children participated in the conversations.

### 3.2. Results from the Deductive Phase of the Analysis

**Figure 1:**
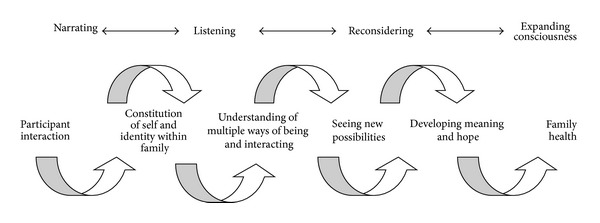
Family Health Conversations illustrated as a spiral movement towards family health.

To reach a possible understanding of how the conversations may support family health, we found it useful to consider Newman's theory of health as expanding consciousness, where health is seen as a synthesized phenomenon constituted by disease and nondisease. Further, health is seen as the “larger whole” and disease and nondisease as reflections of this “larger whole” [7]. From this viewpoint, it appeared to us that the working mechanisms of the FamHCs may be understood as facilitating a spiral movement towards placing family nondisease (i.e., family health) in the foreground. This movement was driven by the verbal interactions between family members and the conversational leaders. It facilitated families' narration and exploration of their experiences, a process in which family members developed an increased understanding of themselves and others and of their interactional patterns. Newman's health theory may also help us understand the importance of this increased understanding in relation to health. She defines nondisease and disease as explicit manifestations of the individual's underlying interactional pattern embracing the individual's interactions with the environment and recognizes a movement toward nondisease as a movement towards an expanded consciousness of this underlying pattern. To further understand this interpretation of the conversations as supporting health, we focused on possible meanings of what could be considered as essential factors in the process, interactional narrating, listening, and reconsidering (see Figure 1).

The starting point of the conversations was the invitation to the family members to narrate their experiences of the family's situation living with illness. Narration has previously been linked to well-being from an individual perspective and may be understood as essential for self-identity and for the understanding of one's experiences. According to Ricoeur [31], narration contributes to the constitution of the self and mediates self-understanding. This is made possible through the connection between the “plot” (constitution of action) in the narrative and the identity of the character (constitution of the self), which is constructed in the narrative [31]. In the FamHCs, family members also cooperated in the construction of a collective narrative. From a dialogic perspective, participating in a reflective dialogue with others facilitates the constitution of an identity with the community. This opportunity is opened up in a context where participants share their feelings and are joined in a shared language [29]. The narrating episodes and the construction of a collective narrative within the FamHCs may thus be seen as a way to increase individual as well as family well-being by facilitating the constitution of a self and support identity-building within the family.

The major difference of family system interventions compared to interventions directed towards one individual is the possibility to bring forth family members' different perspectives. In the FamHCs, family members were invited to listen to other members' experiences and reflections. This offered an opportunity to become aware of multiple ways of being in the situation and of alternative ways of interacting. From a systemic standpoint, information about differences makes a difference to the system [6] and from a therapeutic point of view this is regarded as a ground for change and plausible solutions to problematic situations [32]. According to the Illness Beliefs Model family beliefs are assumed to be connected to suffering and healing, where some beliefs may be facilitating and others constraining in relation to family health. Beliefs are challenged and refined in interaction with others and a dialogue context is seen as a powerful way for a change in beliefs to take place [16]. The FamHCs may have offered the families a context for improving family health by making various beliefs visible and by linking beliefs to family members' different experiences, a process that may have facilitated changes in constraining beliefs.

Narrating and listening seemed essential for reconsidering experiences in the following process. Reconsidering in a dialogic form may have offered an opportunity for families to find new options and develop meaning and hope. Meaning-making has been regarded as a “relational activity,” where meaning is generated and transformed in the response and reresponse from different voices in a dialogue [29]. We found that a new communal understanding of the family's experiences was developed in the process of reconsidering. The meaning of this shared understanding could possibly be understood through Marcel's philosophy of hope [33, 34]. The development and experience of hope is closely linked to intersubjectivity and the establishment of a* we*, which in turn is grounded in “the sharing of concrete, lived experiences” [34, s.234]. In the FamHCs, family members' sharing of their experiences and reconsidering may have improved family health by facilitating meaning-making and development of hope.

The families' narrating, listening, and reconsidering were undertaken in interaction between the participants in the conversations. We interpreted the verbal interactions as moving the process towards family health. The conversational leaders' interactions with the families were found to differ in the two episodes identified. This difference in interactional patterns may be understood in relation to therapeutic interviewing [32], where various types of questions are linked to their different intentions: explanation of problem and revealing of current patterns, or development of insight. Building on the narrating episodes, conversational leaders and families interacted so that explanation and insight were developed in the exploring episodes.

## 4. Discussion

The findings in our analyses indicate that the FamHCs have a theoretically grounded potential to facilitate a movement towards family health. This theoretically driven argument is supported in qualitative studies of family systems nursing interventions when evaluated by families living with chronic illness in various phases [17, 20, 23]. In an integrative review of family responses from participating in systems nursing interventions only a few studies were found indicating that families did not benefit compared to standard care [35]. In another study, six families living with different cancer illnesses in palliative phase described moments during the conversations as being emotionally demanding, although the overall experience was that of a healing and comforting experience [17]. The invitation and facilitation of families' narrating were found to be a starting point for the conversations analyzed in this study. Narrating was also interpreted as an essential part of a movement towards family health. The invitation to tell the family illness story has previously been related to unburdening oneself and as a way for making sense of suffering and finding hope from a family perspective when living with cancer illness in palliative phase [17]. In an interpretative research synthesis study with the aim to develop an understanding of how narratives may be a path to health, the analyses resulted in a model where narrative understanding in a caring conversation was seen as consisting of three phases. The first phase involved the patient telling their story, the second was about narration of the suffering experience, and, in the third phase, the narrative was reconnected to the patient's life story. Going through this process meant going from understanding to interpretation and finally to creation of meaning in and of suffering in connection with illness [36].

The analyses showed that families coconstructed a collective narrative grounded in the individuals' various experiences. In addition family members' understandings of various situations were reconsidered and sometimes altered so that a new communal understanding of the families' experiences was developed. Cybernetic theories imply the importance of viewing individual family members' behaviors as interactional in their nature since feedback is continually received from others. One family member's actions will inevitably influence the behavior of the others and vice versa. This could also be expressed as circular causality where forces in the family move in a circular fashion, implying that it is meaningless to search for* the* cause of an interpersonal event [4]. Consequently, no individual is to blame for a problem experienced within the family [18]. One qualitative study including 16 families living with HIV/AIDS showed that one response to family nursing systems interventions is an increased understanding of family dynamics which opens up for change and contributes to the families' health experiences and individual well-being [21].

The results of the analyses in this study have to be considered in relation to the characteristics of the participating families, the methods chosen, and the preunderstanding of the researchers. The included families varied according to family structure, age, type of chronic illness, time living with illness since onset, and reason for participating (i.e., self-referred or participating in a research project). These variables could have had an impact on the conversations; however, no such differences could be distinguished in relation to what was brought up or to the participants' interactions. In some of the conversations a pattern was more easily distinguished or more or less dominating although it could be found in all of the conversations. This could be seen as strengthening the findings but you have to bear in mind that focus for the study was not to detect differences across families but to see common patterns in the conversations. One might argue that five families is a small sample size and thereby might jeopardize the credibility of the study. However, this choice must be considered in relation to the process of obtaining in-depth knowledge. Data was comprehensive and consisted of 15 conversations, each approximately one hour long, allowing for an analysis that brought forth greater understanding in a circular and reflexive process moving towards a validated interpretation. The inductive analyses built on the first author's (Carina Persson) distinctions of the included conversations. According to Bateson [6], we see certain things as distinctions from the background. There are always many different distinctions and there is always more to see. At the time of the inductive phase of the analyses, the first author was not familiar with conversational analyses or the theories and models that the intervention model is based upon. This opened up for an inductive and an “outsider” perspective which also limited what could be seen. To minimize the risk that essential dialogue events and interactional patterns were overlooked, the findings were discussed with colleagues at the family focused unit at the university and with the second author (Eva Benzein) who has expert knowledge and is skilled in practicing the model. In an attempt to find the most possible understanding of data, the first author strived to become involved in a critical dialogue with the text by making assumptions and actively searching for alternative ways of understanding. In this process, the emphasis was on reaching an understanding characterized by a coherent relationship between the parts and the whole. However, it should be emphasized that the choice of the literature in the deductive phase of the analyses is critical for the understanding of the conversational events as being supportive for family health. The choice was guided by the inductively generated findings and also by the authors' preunderstandings. The findings should therefore be considered as* one* possible way of understanding the potent working mechanisms inherent in the conversations.

## 5. Conclusions

The results from this study offer a description of one model for family systems nursing interventions and, additionally, a theoretically grounded interpretation of how this intervention may support family health. The interpretation showed that narrating, listening, and reconsidering in interaction may be crucial parts in the model. This type of knowledge can hopefully contribute to the successful implementation of family systems interventions in education and clinical practice, with the aim to meet the overlooked needs in care of families experiencing illness.

## Figures and Tables

**Table 1:** Characteristics of the families (*n* = 5).

Type of illness (*n*)	
Stroke	2
Parkinson's disease	2
Dementia	1
Members participating in the conversations (*n*)	
Husband and wife	3
Husband, wife, and children	2
Time since onset of illness (months)	2–60
Family member with illness	
Female (*n*)	3
Male (*n*)	2
Age (range)	40–65
Employed (*n*)	5
Sick-leave (*n*)	1
Retired (*n*)	0
Other participating family members	
Female (*n*)	4
Male (*n*)	5
Age (range)	7–65
Referral to FamHC (*n*)	
Self-referred	3
Participants in one research study	2

**Table 2:** One example of the analysis process of a narrating event.

*What precedes the event?* An exploring event		
Text segment	*What is characterizing this event? *	*Could it be in any other way? Similarities and differences compared to other identified events? *
Woman with illness: I can feel miserable, really low…. because we talk about my illness and my situation but the ones that are close to you…they can of course feel just as miserable…living together with a person that is like a wreck	The woman with illness initiates a new issue that is bothering her.	The event is similar to other events in that a new issue is introduced by a family member following an exploring event. A comparison to other events also shows that new issues are introduced by all partaking family members and also can follow from a narrating event.

Conversational leader A: mm	The conversational leader A is mm-ing	The event is similar to other events in that one of the conversational leaders is mm-ing when a narrating event is introduced by a family member. A comparison to other events also shows that the conversational leaders pose open questions to initiate a narrating event.

Woman with illness: and I think, I think the ones that are next to you and are healthy they have a hell. It's just that…I can feel sorry for my husband, I can feel sorry for everybody that is next to a person with this illness	The woman with illness reflects over the same issue	The event is similar to other events in that the family member who introduces the narrating event goes on reflecting on the same issue. Standpoints and feelings are expressed. A comparison to other events also shows that occasionally the initiating family member hands over the narration of an overwhelming event to another family member.

Conversational leader A: mm	The conversational leader A continues to mm-ing	The event is similar to other events in that the conversational leaders only make short comments. A comparison to other events also shows that the conversational leaders pose follow-up or clarifying questions is necessary.

Woman with illness: nobody asks him: how are you? Are you ok today? Are you happy today? No one asks… he is healthy, yes he is. And that is great… but he is also a human being	The woman with illness goes on reflecting over the same issue	The event is similar to other events in that the family member who introduces the narrating event goes on reflecting on the same issue. Standpoints and feelings are further expressed.

Conversational leader A: mm, but I think that you (turns to the male partner) have expressed how you experience your wife's illness and that the illness have a considerable impact on your situation	The conversational leader A offers her reflection on the issue to the male partner. Her reflection is grounded in what the partner has previously narrated	The event is similar to other events in that one of the conversational leaders turns to other family members to get hold of their standpoints and experiences.

Male partner: yes	The partner agrees with the conversational leader A	The event is similar to other events in that the family member that is invited takes the opportunity and gives his/her response. A comparison to other events also shows that sometimes the other family member has an opposing statement.

Woman with illness: yes, it does, I can…I can feel, it must be a burden	The woman with illness agrees	The event is similar to other events in that the family member initiating the narrating event continues to be active and responds to the other family members' statements and reflections. A comparison to other events also shows that the other family member who was invited by the conversational leader can go on with his/her reflections on the issue.

Conversational leader B: mm	The conversational leader B enter into the episode and is mm-ing	The event is similar to other events in that the conversational leaders takes turns in mm-ing. A comparison to other events also shows that the first time the other conversational leader enters into the episode it can be with a reflection or posing a clarifying or follow-up question.

Woman with illness: it is a heavy burden, I hate this illness. Why I, why him, why we?	The woman with illness goes on reflecting and also introduces a related issue by posing questions	The event is similar to other events in that the family member who initiates the narrating event continues to be active and goes on expanding his/her reflection. A comparison to other events also shows that another way is for the initiating family member to go on and reflect further on the same issue.

Male partner: yes but, you can't think of that. It want take you nowhere. It is like it is…	The partner answers with an opposing statement	The event is similar to other events in that another family member enters the conversation and adds his/her standpoints. A comparison to other events shows that other family members can agree and confirm the narrating family member's standpoints or experiences.

Woman with illness: no, it want take you anywhere	The woman with illness agrees with her partner	The event is similar to other events in that family members respond to other family members' statements or reflections. A comparison to other events also shows that the family member who initiates the issue may not agree but expresses a contradictory standpoint or experience.

Male partner: the question is, how can you do the best of this situation	The partner poses a question in agreement with what he said earlier	The event is similar to other events in that family members continue to reflect on the standpoint they brought up. A comparison to other events also shows that family members can respond to other family members' statements or reflections.

Conversational leader B: yes	Conversational leader B agrees	The event is similar to other events in that the conversational leader only makes a short statement. A comparison to other events also shows that the other conversational leaders also may pose a clarifying or follow-up question.

Woman with illness: yes, the best is to try to understand…I understand you and you understand me. There is no other way to do it.	The woman with illness suggests how to handle the issue in line with her partner's suggestion and comes to a conclusion	The event is similar to other events in that the initiating family member continues to reflect and comes up with a “solution”. A comparison to other events also shows that the initiating family members may not agree but express a contradictory statement or reflection which is followed by a discussion among members.

Conversational leader B: in what way do you try to understand each other?	The conversational leader B poses an exploring question	The event is similar to other events in that either of the conversational leaders poses a question that opens up for an exploration of the issue brought up by the family.

*What follows the event? *An exploring event of the issue that has been narrated		

**Table 3:** Example of one narrating and exploring event in one Family Health Conversation.

Event	
Narrating	CA: Is there something that you would like to bring up in the conversation today?
	Primary narrator: Yes, people invite you to their homes and you wish to go
	Conversational leader A: mm
	Primary narrator: and you think that you have to be there in time, but this illness…
	Conversational leader B: mm
	PN: you can start in the morning and make plans for the rest of the day and at evening, you have done nothing
	CB: mm
	PN: you are not able to finish it
	SN: it's terribly hard for example, if I start to remind you at eleven o'clock that we are supposed to be ready at three
	CA: mm
	SN: we will not make it to three anyhow
	CA: no
	PN: you can, you think, today I will do that and that
	SN: then she finds out other things to do instead of focusing on what needs to be done. So I say, only do what you should and nothing else. And it doesn't work

Exploring	CB: has it always been like this?
	PN: no
	CB: is it something that has appeared now?
	PN: yes, before, I managed to do everything
	CB: yes, but you have always had many things going on simultaneously?
	PN: yes of course, I used to have many things going on simultaneously but suddenly... Now days, when I wish to do something then, I just do it. But I don't get more than halfway and it looks like a total mess but you are supposed to be on your way somewhere. And you got so tired just thinking about it. You become exhausted, thinking about your failures all the time
	CA: yes, do you demand too much of yourself?
	PN: yes… I don't know
	CA: that you should be able to do as many things as before?
	PN: yes I think so, you can't but you do that…you can't but I do
	SN: yes and that becomes distressing. And she has, at times…sometimes she is blocked, she can't move, and sometimes she becomes hyperactive with tremor and twitching
	PN: yes, maybe I should, just as you say, accept everything

CA/B: conversational leader A/B; PN: primary narrator; SN: secondary narrator.

**Table 4:** Categorization, characteristics, and examples of questions asked by conversational leaders in the exploring episodes.

Type of questions	Characteristics	Examples
“Comparative questions”	Focus on exploring change related to situation, function, behavior, feelings, and thoughts and on comparisons and differentiation of various experiences.	In what way do you think that it has changed? What will be the biggest change for you?

“Analyzing questions”	Focus on exploring how interactions, situations, experiences, beliefs, changes, needs, behavior, and reactions manifested themselves and how they were understood and experienced.	How/what do you think then? What does that mean to you?

“Connecting questions”	Focus on exploring the link between two phenomena expressed in the narration not previously connected. Experiences were linked to beliefs or the illness situation. Changes in emotions or behaviors were linked to changes in illness experiences.	Do you think that … makes a difference to …? What you have just said… could this be related to …?

“Concluding statement questions”	Comprised conclusions of what had previously been explored and expressed, now reformulated as a question to the family.	So it was … (conclusion)? It seems like (conclusion)…?

“Interpretive questions”	Comprised nurses' interpretations of meanings grounded in what had been narrated and now presented to the family in the form of a question.	Do you feel that … (interpretation)? Could it be that … (interpretation)?
